# Development of the Mouse Dermal Adipose Layer Occurs Independently of Subcutaneous Adipose Tissue and Is Marked by Restricted Early Expression of FABP4

**DOI:** 10.1371/journal.pone.0059811

**Published:** 2013-03-26

**Authors:** Kamila Wojciechowicz, Karl Gledhill, Carrie A. Ambler, Craig B. Manning, Colin A. B. Jahoda

**Affiliations:** 1 School of Biological and Biomedical Sciences, Durham University, Durham, County Durham, United Kingdom; 2 Biological Stress Response, The Netherlands Cancer Institute, Amsterdam, North Holland, The Netherlands; University of Munich, Germany

## Abstract

The laboratory mouse is a key animal model for studies of adipose biology, metabolism and disease, yet the developmental changes that occur in tissues and cells that become the adipose layer in mouse skin have received little attention. Moreover, the terminology around this adipose body is often confusing, as frequently no distinction is made between adipose tissue within the skin, and so called subcutaneous fat. Here adipocyte development in mouse dorsal skin was investigated from before birth to the end of the first hair follicle growth cycle. Using Oil Red O staining, immunohistochemistry, quantitative RT-PCR and TUNEL staining we confirmed previous observations of a close spatio-temporal link between hair follicle development and the process of adipogenesis. However, unlike previous studies, we observed that the skin adipose layer was created from cells within the lower dermis. By day 16 of embryonic development (e16) the lower dermis was demarcated from the upper dermal layer, and commitment to adipogenesis in the lower dermis was signalled by expression of FABP4, a marker of adipocyte differentiation. In mature mice the skin adipose layer is separated from underlying subcutaneous adipose tissue by the *panniculus carnosus*. We observed that the skin adipose tissue did not combine or intermix with subcutaneous adipose tissue at any developmental time point. By transplanting skin isolated from e14.5 mice (prior to the start of adipogenesis), under the kidney capsule of adult mice, we showed that skin adipose tissue develops independently and without influence from subcutaneous depots. This study has reinforced the developmental link between hair follicles and skin adipocyte biology. We argue that because skin adipocytes develop from cells within the dermis and independently from subcutaneous adipose tissue, that it is accurately termed dermal adipose tissue and that, in laboratory mice at least, it represents a separate adipose depot.

## Introduction

With the upsurge in interest in adipose tissue, in relation to disease and as a source of stem cells, attention is beginning to focus on differences between adipose depots and their developmental origins. Much remains to be clarified about the precise origin and developmental progression of adipocytes [1],[2],[3],[4],[5]. Conventionally, it was thought that many adipocytes develop from a mesodermal lineage, and derive from so-called adipoblasts, early precursors, which differentiate from mesenchymal stem cells [5]. There is also strong evidence that some adipocytes are neural crest in origin [3] and the prevailing view is that white adipocytes in the cephalic region develop from the neural crest, while adipocytes from the trunk area most likely have a mesodermal origin [4],[5]. However, there are many unanswered questions, relating to the number and types of adipocyte precursors; and about the very significant interactions and possible lineage connections between adipose stem cells and their immediate vascular partners [5],[6],[7]. Crucially, adipose depots that have different developmental origins and anatomical locations appear to have varying functional properties, making detailed study of these separate “mini-organs” [5],[8] increasingly necessary.

### Adipogenesis in Skin

In many species subcutaneous adipose depots develop from groups of cells or so called primitive adipose cell organs [9]. In humans, the first evidence of adipose tissue is observed in the second trimester of gestation and “mesenchymal lobules” are seen to develop into “primitive fat lobules” (first signs of lipids) and then to “definitive fat lobules” in which cells contain lipid [10]. Seminal investigations of the development of skin adipose tissues have been carried out by Hausman and colleagues, particularly in pigs e.g. [11],[12],[13],[14],[15]. These animals develop multiple layers of subcutaneous adipose tissue [16], and the extensive studies from the Hausman laboratory have pointed to three important recurring features: the close association between development of the outermost adipose layers and developing hair follicles; differences between this outer layer of adipose tissue and other subcutaneous adipose tissue at the histochemical level; and the spatio-temporal correlation between adipogenesis and blood vessel formation [9],[12]. In rodents, Hausman *et al* [17] studied the development of the adipose layer in newborn rat skin. Prior to adipose formation they observed a looser arrangement of cells in the lower dermal compartment, which they termed the hypodermis. The authors noted connections between adipogenesis and hair follicles; in particular they observed the appearance of lipid containing cells immediately around the bases of the follicles, and noted that the pattern of adipocyte initiation followed that of hair follicle development. They concluded that the skin adipose layer originated from cells immediately surrounding the follicles. They postulated that the process of forming the adipocyte layer involved cells from the upper dermis being translocated down into the hypodermis with the growing/descending follicles.

Recent interest in subcutaneous adipose tissue has been heightened not least because it has been shown to be “beneficial” [18] and also since subcutaneous adipose tissue is a major source of adult stem cells with important translational potential [19],[20]. As in many areas of biomedicine, the laboratory mouse is the default mammalian model system for studying, cellular, molecular and physiological aspects of the adipose organ. Moreover, the relationship between adipose tissue and hair follicles in adult mouse skin has long been a source of interest [21] principally due to the dramatic changes in thickness of the adipose layer in harmony with the hair growth cycle [22],[23],[24],[25]. In mice, hairs grow in waves over the body, making changes to the hair follicle cycles relatively easy to follow. When follicles are actively growing hair fibres (anagen phase), the adipose layer is at its thickest. Subsequently, the follicles regress and shorten in a phase termed catagen, and the period in which fibre growth is turned off completely and which corresponds to the adipose layer in skin being thinnest, is termed telogen. More recently, researchers have started to identify molecular links between adipose tissue and the mouse hair follicle cycle [26],[27]. Others have investigated molecular signatures during adipogenesis in skin identifying several mature adipocyte markers including *Cebpα, Fabp4*, *Pparγ*, adiponectin and adipsin in developing mouse skin [28]. Nevertheless, with the exception of a study on postnatal rats [17], relatively little has been reported on the detailed progression of adipocyte development in rodent skin. We previously observed that pre-adipocyte cells in the lower dermis were marked by CEBPα labelling prior to birth [29] and this heightened our interest in both the origin of the skin adipose tissue and the adipocyte/follicle interrelationship.

Given the importance of mouse models, the present study was designed to elucidate the process of adipogenesis in the skin adipose layer in the mouse. In particular, it was directed at establishing a precise timing of events, further elucidating the relationship between adipocyte and hair follicle development, and determining the immediate origin of the cells undergoing adipogenesis. It was also aimed at finding an early marker of pre-adipocyte tissue. In adult mice, subcutaneous white adipose tissue is present in the interscapular region around the neck and shoulders, and in the inguinal region. However, adipocytes in mature dermal adipose tissue are divided from the subcutaneous depots (beneath the skin) by a muscle layer [1]. In most literature however, no distinction is made between different “subcutaneous” adipose layers in mouse skin. Consequently, a key objective of studying the adipogenesis in skin was to establish definitively where skin adipocytes derive from and whether they develop discretely and independently of the underlying subcutaneous depots?

## Materials and Methods

### Ethics Statement

This study was carried out in strict accordance with the regulations and protocols of the UK A(SP)A [Animals (Scientific Procedures) Act] 1986 and carried out under Home Office Project Licence number 70/7415 granted to CABJ. The protocol was approved by the Ethical Review Panel at Durham University. All surgery was performed by Personal Licence Holders under general anaesthesia (isofluorane), and all efforts were made to minimize suffering including the provision of local anaesthesia (lidocaine/EMLA) and an analgesic (buprenorphine) prior to the operation.

### Origin, Maintenance and Breeding of Mice

CD1 and FVP-GFP mice were from colonies in the Durham University Life Sciences Support Unit. Foxn1^rnu^ mice were obtained from Harlan Laboratories (Bicester, UK). Animals were housed at a temperature of 21°C, with 55% humidity and kept on a 12 hour light/dark cycle. All animals were on Harlan Teklad 2014 Rodent Maintenance diet, and watered and fed *ad libitum*. Analysis of vaginal plugs was used to establish the baseline age of embryos used for experiments. Breeding pairs were kept together overnight and the presence of a vaginal plug next day indicated a successful mating, at which point embryos were considered to be at 0.5 days gestation. Animals were killed using CO_2_/cervical dislocation.

### Sampling of Mouse Back Skin for Lipid Analysis

Skin samples were taken from the backs of (CD1) mouse embryos between e15 and e19, and from post-natal mice from 0.5 to 25 days. To investigate adipocyte development along the caudal–rostral axis, incisions were made in the dorsolateral skin along both sites of the animal from the anterior to posterior direction. Then, skin was cut along the neck and just above the tail and rectangular pieces were carefully removed while preserving the two adipose depots beneath the skin, located towards the head and tail regions. The back skin samples were then embedded in mounting medium (Tissue-Tek^R^ O.C.T.™ Sakura) snap frozen in liquid nitrogen, and stored at −80°C.

### Isolation, Culture and Ectopic Growth of Isolated Mouse Back Skin

Small pieces of dorsolateral skin (∼1 × 2 mm^2^) were microdissected from the same region of CD1 or FVB-GFP mouse embryos at e14 to e14.5. Athymic (Foxn1^rnu^) adult male mice were anaesthetized with isofluorane, provided with analgesic (buprenorphine) and 4 wild type skin specimens were grafted to the kidney capsule of an athymic mouse. A further 6 samples (three GFP and 3 wild type) were transferred to organ culture for 24 hours as previously described [30] before being grafted to the kidney capsule of two mice. Skin samples were imaged using a KY-F1030 digital camera (JVC) fitted to a Stemi SVII dissecting microscope (Carl Zeiss) to visualise external follicle structures. Animals were killed 10 or 12 days post-operatively and specimens were recovered and imaged with a LEICA M165 FC microscope to establish GFP localization. Operations were performed under UK Home Office project license number (70/7415), granted to CABJ. Recovered specimens were either embedded directly in mounting medium (Tissue-Tek^R^ O.C.T.™ Sakura), and snap frozen in liquid nitrogen, or fixed in 4% paraformaldehyde in PBS overnight at 4°C prior to OCT embedding and freezing, or paraffin wax embedding.

### Oil Red O Staining

Sections between 7 and 12 µm thick were cut on a cryostat (LEICA CM 3050S) and attached to SuperFrost^R^Plus slides (Menzel-Glaser), air dried for up to 90 minutes at room temperature (RT), and then stained with Oil Red O dye to detect the presence of lipids. Sections were washed three times in PBS and fixed in calcium formal: 4% formaldehyde (Sigma) and 1% calcium chloride (Sigma) for 1 hour at RT. Next, samples were incubated for 15 minutes in 60% isopropanol (Sigma) and then stained for 15 minutes with Oil Red O solution (Sigma). Samples were then briefly rinsed in 60% isopropanol, washed thoroughly in water, counterstained in Mayers Hematoxylin solution (Fluka) and mounted under coverslips in Glycergel (DakoCytomation).

### HCS LipidTOX Deep Red Neutral Lipid Stain

9 µm cryosections (LEICA CM 3050S) attached to SuperFrost^R^Plus slides (Menzel-Glaser), were air-dried at RT for 90 minutes and fixed in calcium formal at RT for 1 hour. Following extensive washing with PBS sections were incubated with HCS LipidTOX Deep Red neutral lipid stain at RT for 45 minutes (1∶200, Invitrogen). Nuclei were stained with DAPI (Sigma) and slides were coverslipped with Fluorescence Mounting Medium (Calbiochem).

### Haematoxylin and Eosin Staining

7 µm skin cryosections were air-dried at RT for 90 minutes and nuclei stained with Mayers Haematoxylin (Sigma) at RT for 3 minutes. Blueing was achieved by rinsing in tap water while differentiation was achieved by rinsing in 1% acid ethanol. Counterstaining was achieved by rinsing with eosin (Sigma) for 30 seconds while dehydration was achieved by sequential washing with 95% ethanol, 100% ethanol and Histo-Clear (National Diagnostics). Slides were coverslipped with DPX (Agar Scientific).

### Immunofluorescence Staining

7 µm cryosections attached to SuperFrost^R^Plus slides (Menzel-Glaser), were air-dried at RT for 90 minutes and fixed in acetone (Sigma) for 10 minutes at −20°C sometimes followed by methanol (Sigma) for 10 minutes at −20°C. Alternatively, skin samples were fixed in neutral buffered formalin containing 4% paraformaldehyde (VWR International, Ltd.) for 24 hours. Samples were thereafter rinsed twice with 70% ethanol (Sigma) and stored in 70% ethanol at RT until embedded in paraffin wax (Fisher Scientific). 7 µm skin paraffin sections were dewaxed by sequential washing in xylene (Sigma), 100% ethanol (Sigma), 95% ethanol, 70% ethanol and dH_2_O. Antigen retrieval was performed by boiling with freshly prepared 10 mM Tri-sodium citrate for 10 minutes. Non-specific binding was blocked with 10–20% donkey serum (Sigma) at RT for 1 hour. Sections were incubated overnight at 4°C with the primary antibody (anti-FABP4 1∶50, R&D Systems, anti-GFP 1∶1000, Abcam or anti-trichorhinophalangeal syndrome I (TRPS1) 1∶5000, a kind gift from Prof Angela Christiano’s laboratory (Columbia University; New York; USA) followed by a 1 hour incubation at RT with the secondary antibody (donkey-anti-goat-Alexa488 1:1000 or donkey-anti-rabbit-Alexa488 1:1000, Invitrogen). Nuclei were stained with 4′, 6-diamidine-2′-phenylindole, dihydrochloride (DAPI) (Sigma) and slides were mounted under coverslips with Fluorescence Mounting Medium (Calbiochem) or moviol 4–88.

### Skin Section Preparation for Laser Capture Microdissection

Back skin samples, frozen in Tissue Tek, were cut on a cryostat (LEICA CM3050S) and 10 µm skin sections were placed on PALM Membrane Slides (Nuclease and human nucleic acid free PEN/NF (D), Carl Zeiss Microscopy). In addition, the cryostat was wiped with chloroform and slides were kept under UV for 30 minutes before use. Slides with skin sections were air-dried for 6–10 minutes, and underwent a shortened version of Haematoxylin and eosin staining. Samples were incubated in cold 70% ethanol for 2 minutes and washed in DEPC water. They were then stained for 1 minute in Haematoxylin (Haematoxylin solution prepared according to Mayer, Fluka by Sigma), put into Bluing Solution (Thermo Electron Group) for 1 minute, stained for 10 seconds in EosinY solution (Sigma), and washed in 70% ethanol (1 minute), 96% ethanol (1 minute) and 100% ethanol (Sigma) (1 minute). Finally, skin sections were dried for 3–5 minutes at RT, and used for cell collection on the Laser Capture Microdissection Microscope (PALM MicroBeam Zeiss Microscope, CryLaS, FTSS 355–50 with PALM Robo v3.2 software).

### Laser Capture Microdissection

Skin sections were examined under the Laser Capture Microdissection (LCM) Microscope and two areas of dermis were selected for the subsequent collection of material. Area “1” (upper dermis) was characterized by cells localized in the upper dermis (later validated by the upper dermal marker TRPS1 [31]), close to the epidermal layer, whereas Area “2” (lower dermis) was represented by cells from the lower dermis, between and around hair follicle end bulbs. The specimens from the two areas of dermis were collected in two separate PALM Adhesive Caps (Carl Zeiss Microscopy), 50 µl of lysis buffer RLT (Qiagen) with β-mercaptoethanol (Sigma) was added and the sample was incubated for 10 minutes on ice and stored at −80°C. From a single embryonic sample (for Area “1” or Area “2”) the minimum number of collected cells that was used for RNA isolation was around 3000–9000. In addition, three mouse embryos (from the same mother) were used for each analysed time-point; therefore the analysis was performed in triplicates.

### Quantitative RT-PCR

Total RNA was extracted using the RNeasy Micro kit from Qiagen. The quantity of total isolated RNA was determined on a NanoDrop Spectrophotometer (ND-1000 with V3.5.2. software, Labtech International), and the integrity checked on a denaturing agarose gel. RNA was amplified and cDNA synthesised using the GeneChip Two-Cycle cDNA Synthesis Kit from Affymetrix. All procedures were carried out according to the manufacturer’s instructions under RNase free conditions. Quantitative RT-PCR was performed on a 7300 or 7500 Fast Real-Time System with compatible 7300 or 7500 Fast System software (SDS version 1.4 Applied Biosystems). The “ddCt Relative Quantitation” assay was used for this work. Each individual PCR reaction (20 µl total volume) contained diluted (1∶5 or 1∶10) cDNA, primer mix (2.5 µM forward primer and 2.5 µM reverse primer), Power SYBR Green PCR Master MIX (Applied Biosystems) and sterile water. For each cDNA used, four identical reactions were prepared for each set of primers. Reactions were performed in 96-well reaction plates (Applied Biosystems). The conditions of the quantitative RT-PCR reaction were: step 1 (95°C for 10 minutes) followed by 40 cycles of step 2 (95°C for 15 seconds) and step 3 (60°C for 1 minute). The primer sequences were: *TRPS1* (upper dermal marker) [31] forward 5′TCTCTCTGGCGAAAGAATGC, reverse 5′CCCTCTGCTGTTTGTTGAGC, *Fabp4* forward 5′GATGAAATCACCGCAGACG, reverse 5′GCCCTTTCATAAACTCTTGTGG, Ribosomal protein, large, P0 (*Rplp0)* (housekeeping gene) forward 5′CACTTACTGAAAAGGTCAAGGC, reverse 5′AGACCGAATCCCATATCCTCA.

### Imaging and Image Analysis of Microscopic Skin Samples

Immunofluorescence, histological analysis and imaging was performed on a Zeiss Axio Imager M1 microscope with OpenLab software (Improvison) or a Zeiss 510 Meta Confocal Laser Scanning microscope with corresponding software (Carl Zeiss). To investigate changes in the volume of adipose tissue, and the relative thickness of different layers of postnatal skin, microscopic images obtained as described above, were analysed using Image-J software [32]. Briefly, colour based threshold detection was used followed by the watershed tool. The analyse particles function was used to draw around all red areas and the outlines passed to the region of interest manager (ROI). Next the particles were analysed and stored to the ROI manager where areas due to background and epidermal staining could be manually removed. After this the volume of the remaining areas was measured. An image-J macro was written to rapidly perform these steps and can be found at: http://www.dur.ac.uk/craig.manning/OilRedO_0-4.txt.

## Results

### Features of Back Skin and Skin-Related Adipose Cells during Mouse Development

Macroscopic observations of adipose tissue distribution under the dorsal skin of developing mice confirmed previously reported [1] subcutaneous depots under the neck region, and above the junction of the lower limbs with the spinal cord (data not shown). Rostral to caudal sectioning was then carried out on strips of back skin retaining these subcutaneous depots, at intervals between e16 and postnatal day 25. Oil Red O staining allowed the distribution of adipogenic cells in and beneath the skin to be closely monitored during late embryonic development and through the first hair growth cycle after birth in mouse skin. In mouse back skin the anatomical features that signify the start of a first wave of hair follicle development are seen around e14.5, when guard hairs start to develop. A second wave producing awl and auchene hairs is initiated at e16.5 followed by zigzag hair development at e18.5. When the awl/auchene hairs start to develop at e16.5, guard hairs have already started to grow down into the dermis.

**Figure 1:**
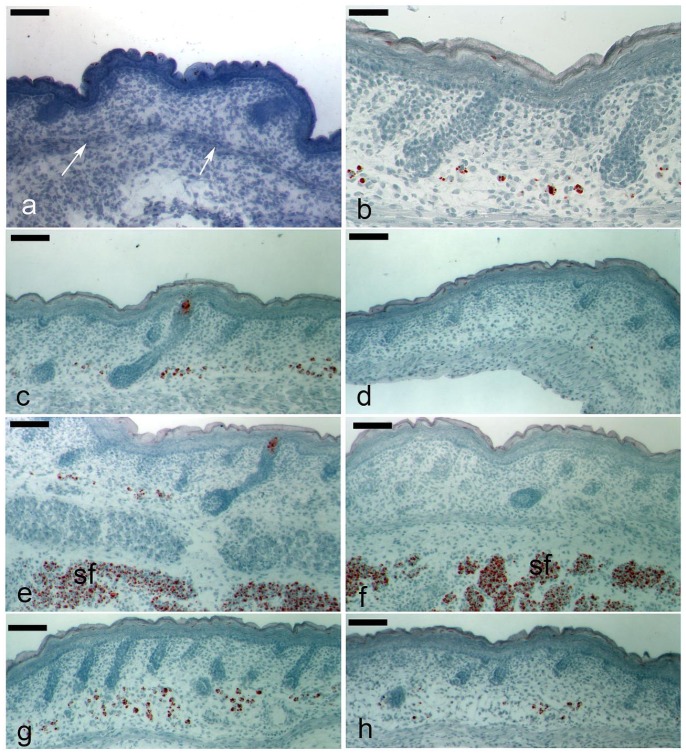
Early accumulation of lipid in cells of the lower dermis follows a spatio-temporal pattern. Samples of dorsal skin from foetal and early newborn dorsal skin sectioned and stained with Oil Red O to detect lipids (a-h). At e16 no lipid accumulation is detected anywhere in skin (a) note the line of cells representing the precursor of the *panniculus carnosus* (white arrows). By e18.5–19 the first lipid containing cells are detected, at which point the lipid is often multilocular (b). Sections from the head (c and e) regions of e18.5 dorsal skin show larger numbers of cells with accumulation of lipid in the deep dermis compared with tail regions (d and f) where far fewer lipid producing cells were apparent. In regions between the head and tail, some areas had consistent groups of cells containing lipid (g). Lipid forming cells are occasionally observed in regions where follicle development is apparently less advanced (h). sf = subcutaneous adipose tissue. Scale bars: a, c, d, e, f, g, h = 100 µm, b = 50 µm.

As reported previously [29] Oil Red O staining was never seen at e16 or e17 in the dorsal skin dermis (Figure 1a). Staining was first apparent around e18.5, and only in the deep or lower half of the dermis around or below the bases of descending guard hair follicles. At this stage lipids within cells were multilocular, with droplets that varied in size but that included some very small inclusions (Figure 1b). Examination along the anterior to posterior axis of cells that had started lipogenesis revealed that their distribution was not uniform. Intriguingly, specimens showed the same pattern in that towards the anterior of the mouse some lower dermal cells had made fat, while approaching the tail, or posterior, dermal cells with stained lipid droplets were only rarely visible (Figure 1c–f). However, this was not a strictly linear distribution since patches of stained cells were also seen in the mid dorsal region (Figure 1g). The simple explanation is of a correlation between start of lipid production and stage of follicle development, which generally lagged in the posterior region. However, on occasions lipid producing cells were observed overlying follicles apparently at a relatively early stage of development (Figure 1h). There was no link between the early appearance of fat and presence of a subcutaneous fat depot, as these were underlying both caudal regions where dermal cells had started to accumulate fat (Figure 1e) and rostral skin where they had not (Figure 1f).

**Figure 2:**
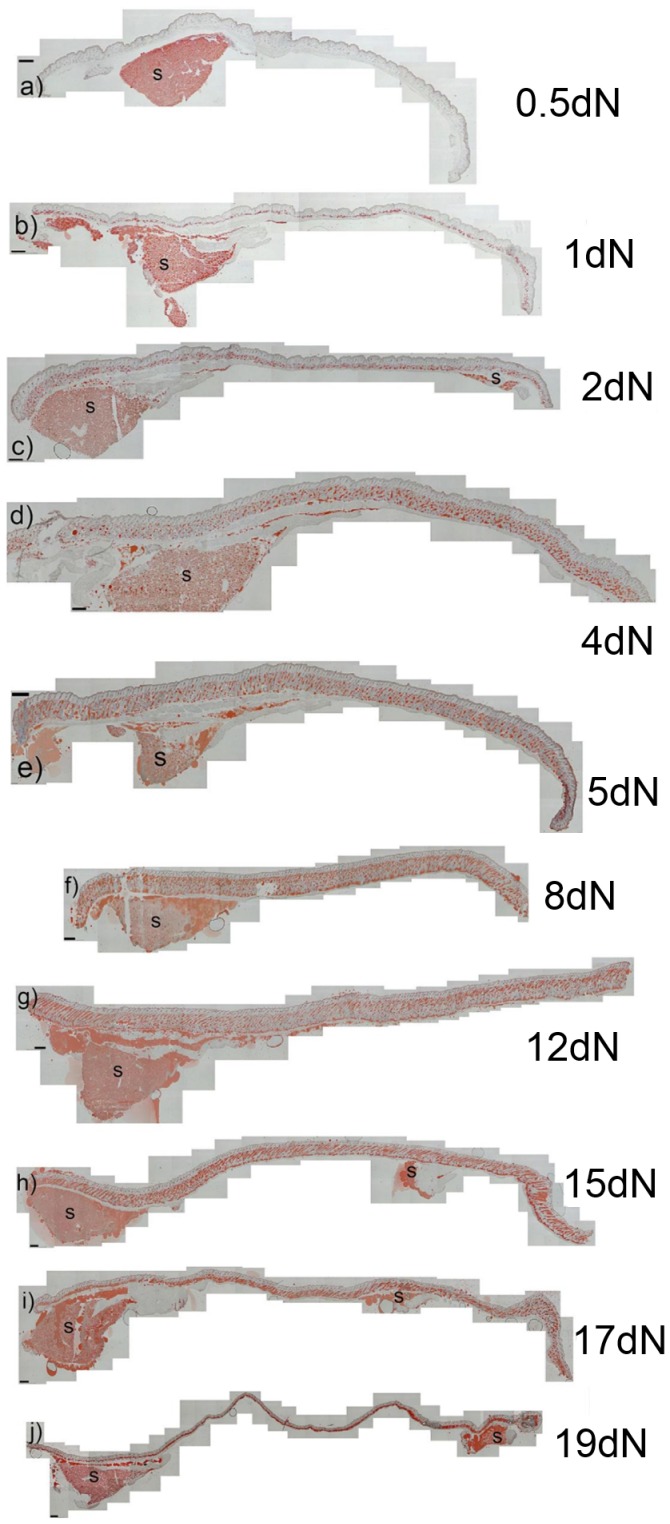
Lipid accumulation in back skin specimens with adipose depots beneath the skin. Composite image of longitudinally sectioned skin section stained with Oil Red O to show lipid accumulation along the anterior-posterior axis. Samples were prepared from newborn mice at different postnatal time points 0.5 day,1 day, 2 day, 4 day, 5 day, 8 day, 12 day, 15 day, 17 day, 19 day. S = subcutaneous adipose tissue below the anterior and some posterior regions of the skin specimens. (a – j) Scale bar = 300 µm.

**Figure 3:**
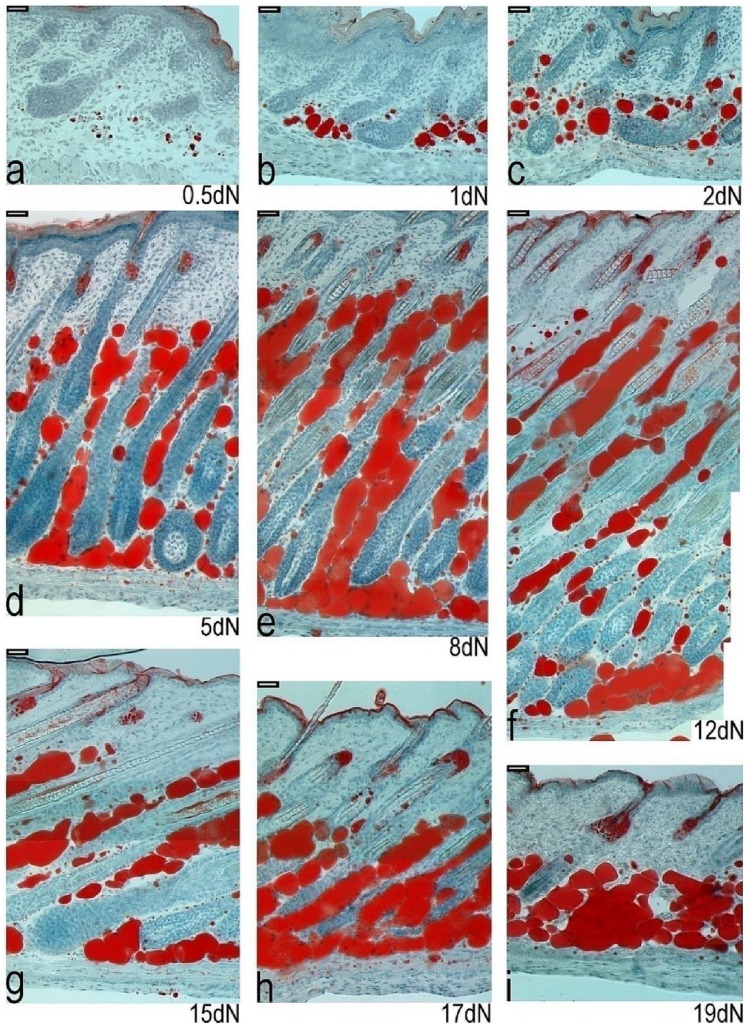
Changes in skin thickness and dermal adipose tissue development in lower skin dermis of the newborn mouse. Skin sections were stained with Oil Red O to detect lipids from birth to 19 days postnatally (a–i). In the 36 hours after the 0.5dN time point (a) there is a rapid increase in both the numbers of stained cells and the size of the lipid droplets (b, c). In association with the downward expansion of follicles the dermal adipose tissue layer thickens until day 12 (f) with the most rapid expansion between about 2 and 5 Days (c, d). As follicles regress, dermal thickness decreases (g, h) until skin thickness is reduced back to that seen in the days shortly after birth, and lipid containing cells accumulate below (i). Scale bar = 30 µm.

Oil Red O stained adipocytes, were still patchily distributed shortly after birth (Figure 2a). In one and two day newborn specimens however, a distinct layer of labelled adipose cells was visible in the lower dermis along the entire length of the rostral-caudal axis (Figure 2b,c). Over subsequent days the depth of the new adipocyte layer progressively and rapidly increased, coincident with the extension and downgrowth of hair follicles and by day 8 follicles were reaching their full downward extension (Figure 2d–f). At 12 days (Figure 2g) the lipid layer was at its maximum thickness, representing 80% or more of the total dermal depth. However, from 15 days with the onset of the catagen stage of the hair cycle the lower dermis was noticeably thinner in the anterior region (Figure 2h) and by 17 days the thickness of the adipose layer had further diminished along the length of the animal (Figure 2i). It reached its thinnest, around 19 days after birth, at which point follicles had regressed to telogen and the adipocyte layer represented about 50% of dermal depth (Figure 2j). By postnatal day 26, when a new hair follicle growth phase (anagen) had started, the adipocyte layer in the dermis was restored to around 70% of the total dermal thickness (data not shown). More detailed examination of the specimens (Figure 3) confirmed these observations, and showed that the rapid early postnatal expansion of the dermal adipose layer was due to a combination of an increased numbers of fat containing cells and an expansion in size of the lipid droplets. A few hours after birth substantial numbers of cells in the lower dermis were still without lipid and those that had fat contained relatively small multilocular droplets (Figure 3a). By postnatal day 1 and 2, however, larger rounded unilocular fat lobules had developed, and a high proportion of cells in the lower dermal compartment had some fat staining (Figure 3b,c). By 5 days the dermal adipose tissue layer mainly consisted of unilocular cells with large fat droplets (Figure 3d). These were initially round in shape, but over time many adipocytes became elongated, or flattened as they filled nearly all the space and became squeezed between and beneath hair follicles (Figure 3e–h). At telogen the adipocytes reduced in size and coalesced in the space vacated by the regressed follicles (Figure 3i).

The above observations showed that the relative thickness of the lower dermis as a proportion of the total depth of dermis increased and decreased concomitant with follicle elongation and shortening in the first hair cycle. Image analysis established that in mid anagen (10 days postnatal) when follicles were fully extended the dermis contained significantly more (over 4 times as much) adipose tissue as when the follicles had regressed to late catagen/telogen (19 days postnatal) (See Figures S1 and S2 and Table S1). At the same comparison time-points we observed no significant difference between the area of the upper dermis (data not shown).

### Cellular Changes Associated with Expansion of the Adipose Layer in the Dermis

**Figure 4:**
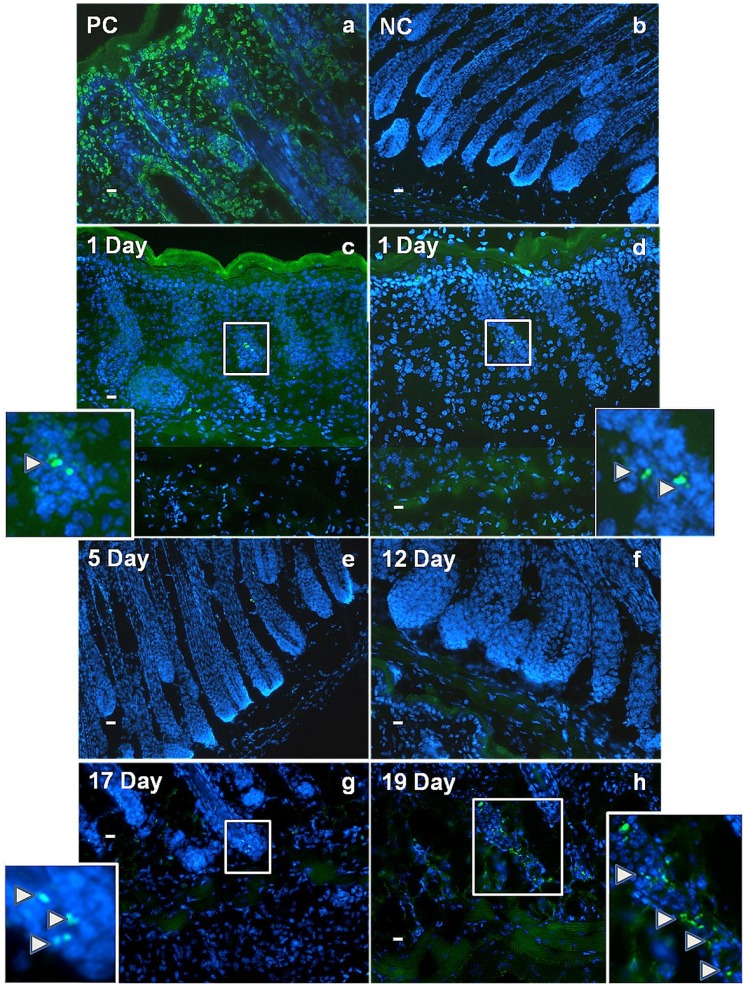
No significant apoptosis is observed in the adipocyte layer, during its formation, or at any point in the first hair growth cycle as measured by the TUNEL assay. TUNEL (terminal deoxynucleotidyl transferase dUTP nick end labelling), a) PC (positive control - nuclease treated 1 day old mouse back skin), b) NC (negative control - 1 day old mouse back skin). In one day specimens labelled cells were occasionally observed in hair follicles (magnified and arrowed in c, d) and in the developing adipose layer (c) but more often they were completely absent from the latter (d). Apart from very occasional follicle-associated labelling virtually no cell death was observed at 5 and 12 days after birth, through the middle stages of the first hair cycle (e, f). Subsequently an increasing amount of cell death was observed associated with regression of the follicles as their growth shut down at 17 and 19 days (magnified and arrowed in g and h) however virtually no cell death labelling was observed in the surrounding adipocyte cells. DNA was counterstained with 4′,6-diamidino-2-phenylindole (DAPI). Scale bar = 30 µm.

A possible explanation for the observed changes in the composition of the lower dermis was that a substantial proportion of the original dermal cells were dying, leaving an expanding adipogenic sub population to populate the space. To investigate this, TUNEL staining was performed on skin sections at regular intervals (Figure 4). During late foetal and early postnatal development (Figure 4c,d) a few TUNEL positive cells were seen in the epidermis, follicle epithelium and dermis, and in the muscle layer, but no evidence was found of significant apoptotic cell death where adipogenesis was occurring in the lower dermis. Through the main growth (anagen) stages of the first hair cycle at day 5 and day 12, virtually no cell death was visible (Figure 4e,f). As would be anticipated, a substantial amount of cell death was apparent in the epithelium of hair follicles at P17 and P19 when follicles were progressing through the regressive (catagen) stage of the hair cycle, but this was only accompanied by very occasional labelled cells in the surrounding adipo/vascular cell population (Figure 4g,h).

### FABP4 is a Marker of the Pre-Adipogenic Lower Dermis

**Figure 5:**
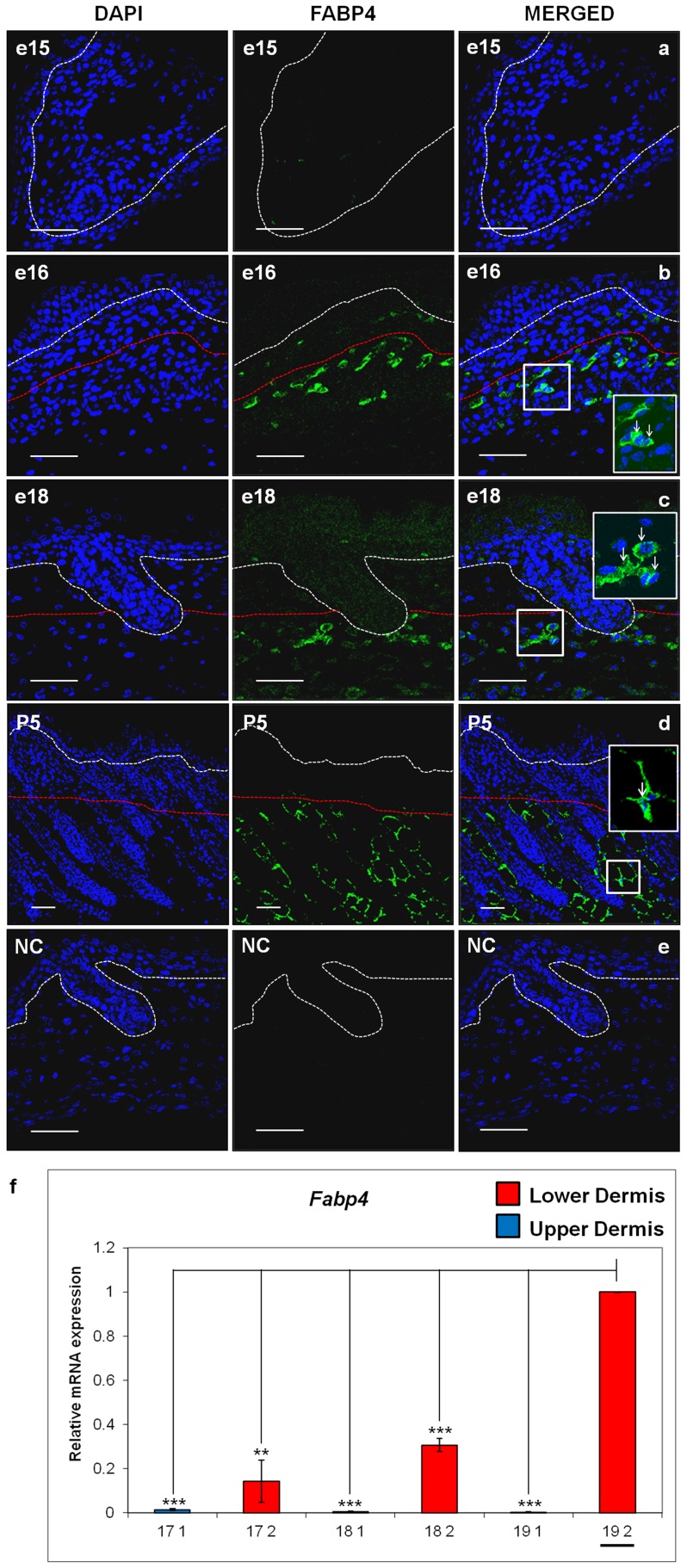
Fatty acid binding protein-4 (FABP4) is expressed in the lower dermis of developing mouse back skin from embryonic day 16 (e16). a), e15 (embryonic day 15), b) e16 (embryonic day 16), c) e18 (embryonic day 18), d) P5 (post-natal day 5), e) NC (negative control - no primary antibody). White dashed line delineates boundary between epidermis and upper dermis. Red dashed line delineates boundary between upper dermis and lower dermis. DNA was counterstained with 4′,6-diamidino-2-phenylindole (DAPI). Scale bar = 50µ m. f) The relative mRNA expression for *Fabp4* was analysed from samples at the e17 (17), e18 (18) and e19 (19) time-points from upper (Area “1”, blue) and lower (Area “2”, red) dermis. The baseline (1-fold change) was established for the e19 lower dermis sample (underlined on the figure) and the mRNA levels of the other samples are shown relative to this. *Fabp4* mRNA is up-regulated in lower dermis (Area “2”) compared with upper dermis (Area “1”) at all analysed time-points. Data were obtained from triplicate biological replicates. P value refers to the comparison with “19 2” sample (*P≤0.05, **P≤0.01, ***P≤0.001).

**Figure 6:**
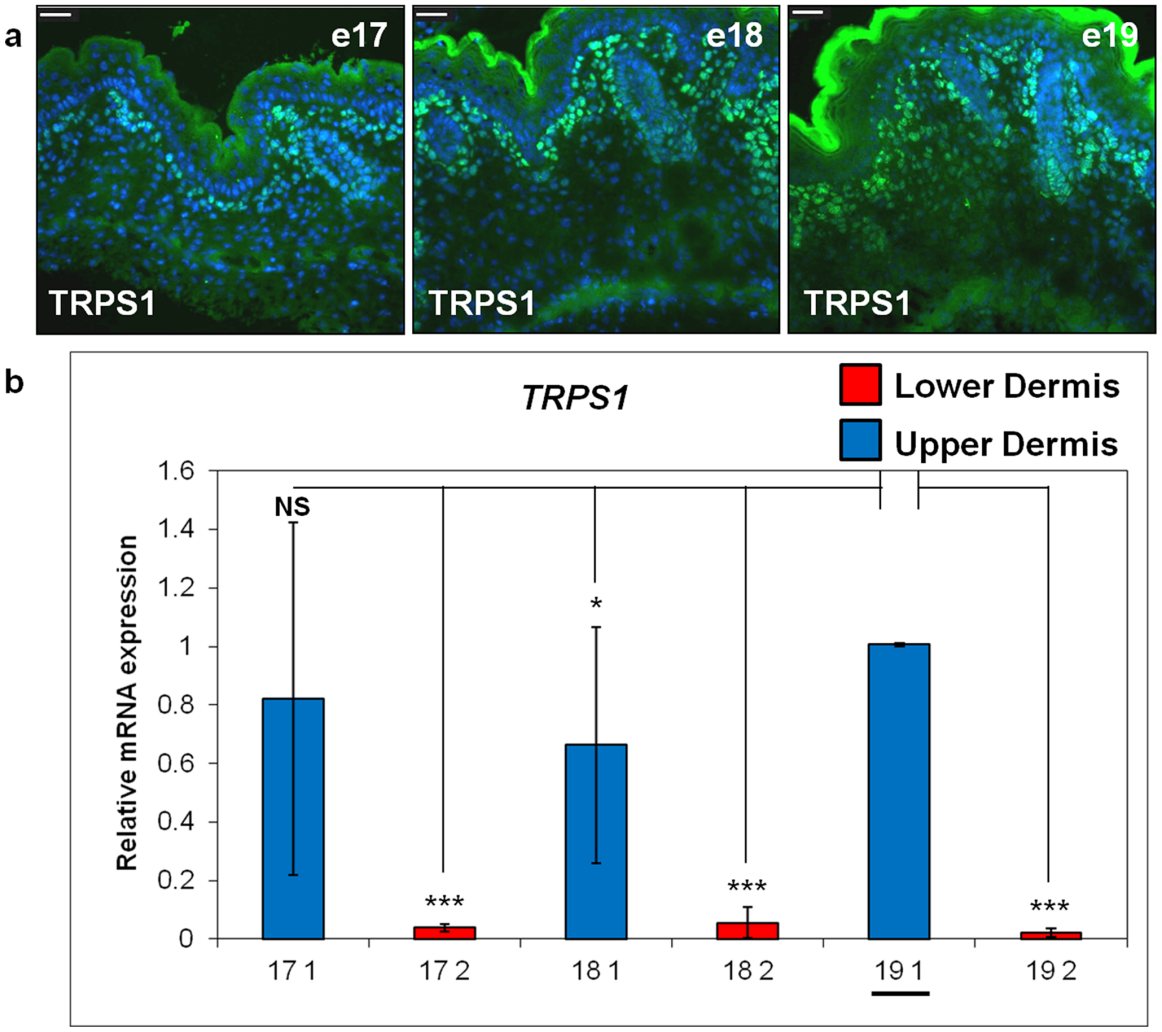
Trichorhinophalangeal syndrome I (TRPS1) expression is restricted to upper dermal cells at embryonic day 17 (e17), e18 and e19 in murine dermis. a), DNA was counterstained with 4′,6-diamidino-2-phenylindole (DAPI). Scale bar = 30 µm. b) The relative mRNA expression for *TRPS1* was analysed from samples at the e17 (17), e18 (18) and e19 (19) time-points from upper (Area “1”, blue) and lower (Area “2”, red) dermis. The baseline (1-fold change) was established for the e19 upper dermis sample (underlined on the figure) and the mRNA levels of the other samples are shown relative to this. *TRPS1* mRNA is up-regulated in upper dermis (Area “1”) compared with lower dermis (Area “2”) at all analysed time-points. Data were obtained from triplicate biological replicates. P value refers to the comparison with “19 1” sample (*P≤0.05, **P≤0.01, ***P≤0.001).

We previously showed that the transcription factor CEBPα was a reliable marker for the nuclei of committed individual pre-adipocyte cells from around e17 to e18 onwards [29]. Now, having established that the bulk of the lower dermis turns into adipose tissue, we aimed to find an earlier marker that would highlight that region of the dermis committed to adipogenesis. The lipid transport protein, FABP4, has previously been shown to stain adipocytes in newborn mouse skin [33]. When we investigated FABP4 expression in mouse skin sections, between e15 and P5, no specific labelling was observed at e15 (Figure 5a). However, by e16 there was distinct intracellular labelling of cells in the lower dermis (Figure 5b). Moreover, widespread marking of cells predominantly of the lower but not the upper interfollicular dermis was observed between e18 and postnatal day 5 time-points (Figure 5c,d) (Figure S3 for additional images). There was little or no labelling of the hair follicles or inter-follicular epidermis (Figure 5a–d) and the negative control sections were also blank (Figure 5e). Additionally, we used quantitative RT-PCR on Laser Captured tissue to confirm that *Fabp4* mRNA expression was overwhelmingly expressed in the lower dermis compared to the upper dermis, and that levels of expression of *Fabp4* in the lower dermal compartment increased significantly between e17 and e19 (Figure 5f). To confirm that we could successfully discriminate between the upper and lower dermis and then isolate tissue from each of them using Laser Capture Microdissection we investigated expression of a known marker of the upper dermis. The transcription factor TRPS1 has been previously shown to be a marker of hair follicle mesenchyme and the upper interfollicular dermis around the time of follicle development [31]. Here we used TRPS1 antibody staining to show that cells in these areas continue to be highlighted in foetal dermis up to e19 (Figure 6a). Moreover, quantitative RT-PCR on tissue captured from upper or lower dermis at e17, e18 and e19 confirmed that *Trps1* mRNA expression was almost exclusively in upper dermis compared to the lower dermis (Figure 6b).

### Interactions between the Dermal Derived Adipose Tissue and Other Adipose Depots

**Figure 7:**
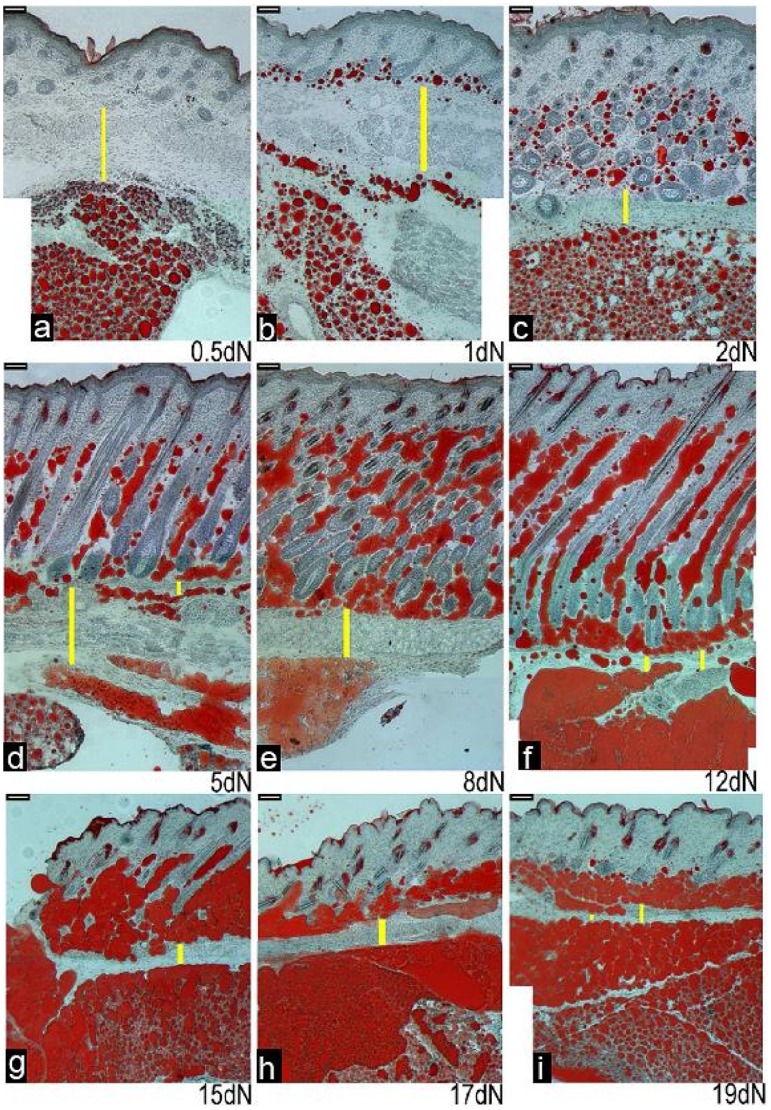
Relationship between developing skin adipose tissue, the *panniculus carnosus* and underlying subcutaneous adipose tissue in the dorsal skin of newborn mice. Mouse dorsal skin sections from the region overlying the anterior (interscapular) subcutaneous adipose depot. Skin sections with underlying subcutaneous adipose tissue were stained with Oil Red O to detect lipids from birth to 19 days postnatally (a–i). The thickness (marked by yellow lines) of the *panniculus carnosus* layer changes in the specimens over time. An additional thin layer of lipid droplets can be seen under the dermal adipose tissue, for example at day 5 (d - see area close to smaller yellow line). a) 0.5 day old newborn mouse. b) 1 day old newborn mouse. c) 2 day old newborn mouse. d) 5 day old newborn mouse. e) 8 day old newborn mouse. f) 12 day old newborn mouse g) 15 day old newborn mouse. h) 17 day old newborn mouse. i) 19 day old newborn mouse. (a - i) Scale bar = 65 µm.

From late foetal development a muscular *panniculus carnosus* layer was visible along the whole length of back skin specimens under the lower dermal cells and adipose tissue (Figure 2a,b). Along much of its length this was a single layer of relatively uniform thickness that did not change significantly from birth onwards. However, in the anterior half of the back skin, around where it covered the subcutaneous adipose tissue the structure of this layer was more complex. It was initially much thicker (Figure 7a), and above the interscapular subcutaneous depot it split and in some areas an offshoot of the subcutaneous adipose tissue was visible between the two muscle layers (Figure 2a,b and Figure 7b). Above the main interscapular subcutaneous depot the thickness of the *panniculus carnosus* generally decreased from postnatal day 5 to 19 (Figure 7d–i). Periodically, in some specimens, lipid containing cells were seen within the *panniculus carnosus* and some of these were close to both the dermal adipose tissue and the underlying subcutaneous layer (Figure 7i). Crucially though, the dermal derived adipose tissue and the established subcutaneous adipose depot did not merge in any part of the skin at any of the time points, therefore, the *panniculus carnosus* continuously separated these two tissue clusters.

**Figure 8:**
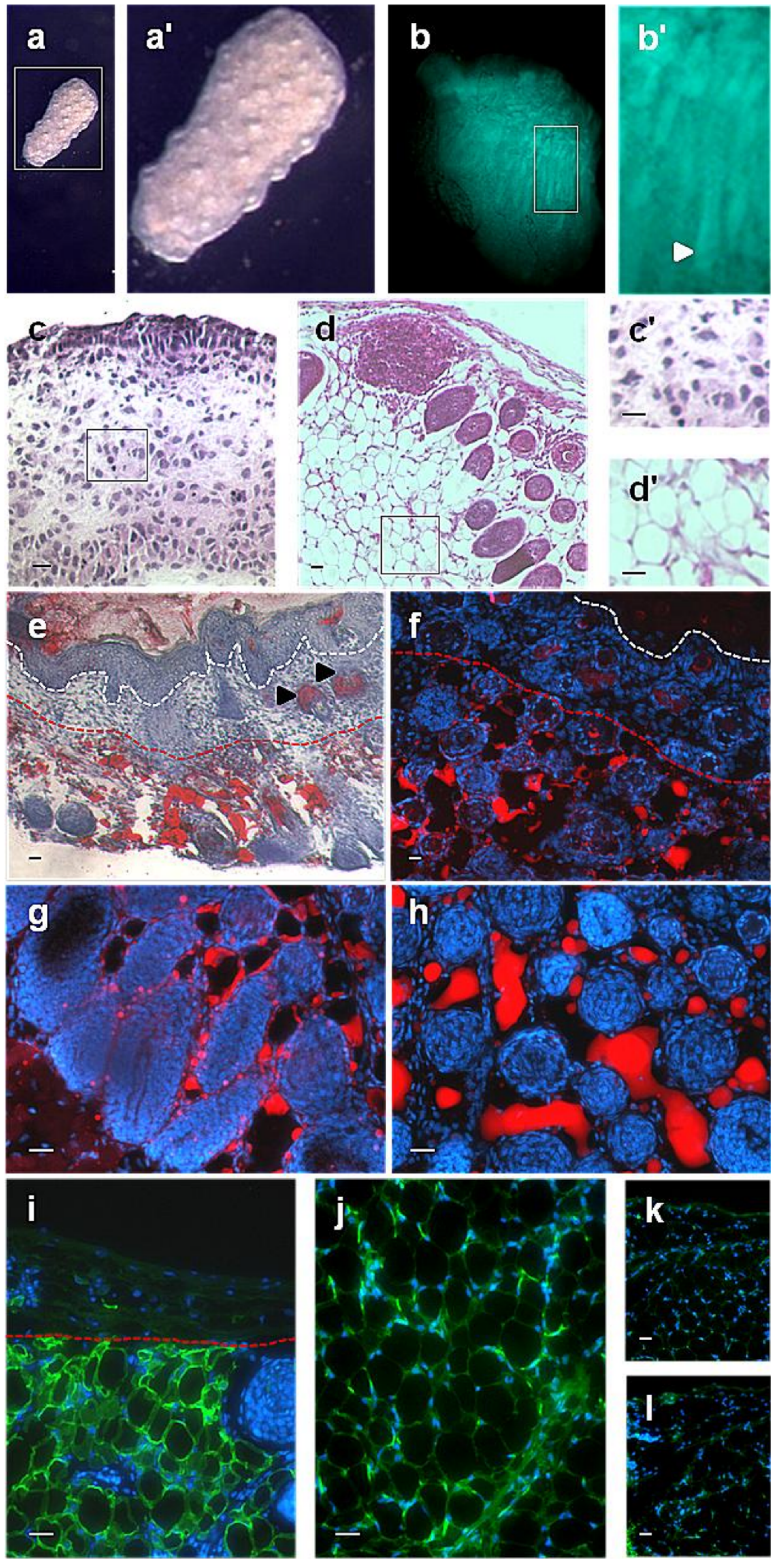
The lower dermis becomes the skin adipose layer independently of subcutaneous influence. External view of green fluorescent protein (GFP)-labelled E14 mouse back skin, microdissected and cultured for 24 hours on agar (a and a′). Fluorescent microscopy of mouse back skin grafts showing presence of GFP 10 days after grafting to the kidney capsule of a non-GFP nude mouse. Both hair follicles and the surrounding adipose tissue express GFP (b and b′) Haematoxylin and eosin (H+E) staining of E14.5 mouse back skin showing absence of skin adipose tissue(c and c′) and 10 days post-grafting to the kidney capsule showing the presence of a skin adipose layer in the lower dermis (d and d′). Confirmation of lipid production only in the lower dermis of skin grafts by Oil Red O staining (e) and lipidTOX staining (f-h). Fatty acid binding protein-4 (FABP4) is highly expressed in the lower but not the upper dermis of grafted skin after 10 days (i) Antibody staining for GFP in the dermal adipose layer of the grafted mouse back skin confirmed donor origin (j). Negative controls, (no primary antibody) for FABP4 and GFP antibody staining, respectively k and l. White dashed line delineates boundary between epidermis and upper dermis. Red dashed line delineates boundary between upper dermis and lower dermis. Black arrowheads in figure e (sebaceous glands). In figures f, g, h, i, j, k and l DNA was counterstained with 4′,6-diamidino-2-phenylindole (DAPI). Scale bar = 30 µm.

These data demonstrated that the two populations, the subcutaneous depot and the adipocytes originating from the dermis, do not coalesce developmentally but remain separate. This did not, however, exclude the possibility that the subcutaneous adipose layer influences, or is required for, the development of adipocytes in the dermis. To investigate this we tested whether skin would undergo the same developmental progression and produce an adipose layer in the lower dermis when entirely separated from any subcutaneous influences. Skin was removed from the interscapular region of wild type and GFP mouse embryos at e14 to e14.5. Some pieces were initially put into organ culture for 24 hours where the pattern of developing follicles became prominent externally (Figure 8a,a′). Specimens were then removed and grafted to the kidney capsule of athymic mice for 10 or 12 days. All of the specimens were recovered although on two occasions two individual specimens had coalesced. The recovered GFP specimens showed fluorescent labelling of follicles and surrounding adipose tissue (Figure 8b,b′). H and E staining established that the skin dermis, which at e14.5 (Figure 8c,c′) does not express the pre-adipocyte marker CEBPα [29] or FABP4 (Figure 5), had developed a lower adipose layer with typical rounded locular adipocyte cells (Figure 8d,d′). This was confirmed by Oil Red O (Figure 8e) and LipidTOX (Figure 8f–h) staining which, apart from patchy staining of sebaceous glands, both showed labelling uniquely in the lower dermis and surrounding hair follicles. FABP4 labelling demonstrated that the expression difference between the upper non expressing dermis and the lower adipocyte forming dermis was maintained after grafting (Figure 8j). Positive staining with a GFP antibody verified that cells of the new adipose layer had originated from the original skin specimens (Figure 8i). As seen in Figure 8k and 8l, there was no non-specific labelling of skin sections with the donkey-anti-goat-Alexa488 or donkey-anti-rabbit-Alexa488 secondary antibodies, respectively.

## Discussion

### Origin and Timing of Adipose Development in the Dermis

Here we describe the events that lead to adipose formation in the dorsal skin of the mouse. We observed that lipid containing cells, often multilocular, first appeared prenatally in the lower dermis around and below the level of penetration of developing pelage follicles. This was followed by dramatic expansion of the lower dermis involving both downward extension of follicles, and further adipogenesis and lipogenesis between 1 and 4 or 5 days after birth. These findings are consistent with published work [17], although we have an alternative preferred interpretation of events. We suggest that pre-existing cells in the lower dermis from well before birth, essentially become the adipose layer. This explanation differs from our initial hypothesis and that previously proposed for the rat, in which the authors propose that follicles translocate pre-adipocytes down into the lower dermis as they elongate, to create the skin adipose layer [17]. While this cannot be ruled out, our evidence points to there being cells in the lower dermis committed to and initiating adipogenic differentiation in the young foetus. In previous studies we have shown that, prior to lipogenesis, adipogenic cells are labelled by CEBPα, and that cells in the lower dermis express this marker from around e17 to e18 [29]. Therefore, as in most species, adipocyte formation is preceded during foetal development by the establishment of pre-adipocytes [9]. Nevertheless, our observations are consistent with the rat studies [17] in finding a strong association between the appearance of lipid in specific regions of the dorsal dermis, and the extent of hair follicle maturity in that area, pointing to some form of linked developmental signalling.

To further investigate the idea of lower dermal cells being “adipogenic” and distinct from the upper dermis, we studied the expression of the adipogenic marker FABP4 in pre-natal skin. Ablation of FABP4 in mice causes early postnatal lethality, and although it does not reportedly affect hypodermal adipose development to this point [34], FABP4 is considered to be a marker of later adipogenic differentiation. Here its expression was strikingly restricted to the lower dermis from as early as e16, well before any lipid accumulation. This provides strong evidence of there being developmental differences between the upper and lower dermis, with the latter undergoing programmed adipogenic transformation. Recent work using array analysis on adipocyte precursors isolated from developing skin of leptin-luciferaseBAC transgenic mice has also reported up-regulation as early as e17.5, of transcripts of markers normally associated with adipocyte development and differentiation including *Cebpα*, *Fabp4*, *Pparγ*, adiponectin, and adipsin [28]. This finding accords largely with our own observations (unpublished) of the early appearance of adipocyte cell markers, and our demonstration at e16 of widespread FABP4 protein expression in the lower dermis is further evidence of the spatial compartmentalisation of the dermis occurring at a key developmental stage. Moreover, we confirmed that at the mRNA level *Fabp4* expression in the lower dermis of foetal mice was increasing over time, corresponding with the progress of adipogenesis in that compartment.

Amongst other cellular events that might accompany the rapid transition from there being a distinct lower dermal compartment to one comprising follicles, adipose tissue and blood vessels was the possibility that a cohort of existing dermal cells were dying. However, when this phenomenon was investigated in this study, no more than minimal cell death was observed in the lower dermal cells at the peak time of their transition into adipocytes, so cells are apparently not apoptosing to create space for an influx of adipocytes.

### The Dermal Adipose Layer is Distinct from Subcutaneous Adipose Depots - Nomenclature and Cellular Origins

The above data suggested that the immediate origin of all the adipocytes in dorsal mouse skin was the dermal layer, however true “subcutaneous” adipose deposits are present beneath both the neck (interscapular) and lower abdominal (inguinal) regions of dorsal skin. As well as deeper brown adipose deposits, white adipose tissue is reported to develop in the interscapular region shortly after birth [35]. Indeed, in the current study, a bifurcation of the subcutaneous adipose deposit was seen, with a layer of larger lipid containing cells extending posteriorly from the main structure. There remained the possibility, therefore, of intermixing of the skin-derived and subcutaneous depots, and interestingly, elegant lineage tracing work has suggested that in dorsal region, interscapular adipose tissue and dermis develop from the same origin, the central dermomyotome [36]. Our anatomical observations showed that the dermal adipocytes did not merge with underlying adipose depots at later developmental time-points, and the two remained separated by the *panniculus carnosus*. More importantly, perhaps, by isolating skin from e14 embryos, before the initiation of adipogenesis and growing them in the kidney capsule, we were able to show that adipocytes develop autonomously in the lower dermis, and that signals from subcutaneous adipose depots are not required for this to occur. This implies that adipose development in skin is controlled by intrinsic cellular and molecular signalling, at least from e14 onwards. This experiment also ruled out the somewhat unlikely possibility of these adipocytes deriving from circulating progenitor cells [37],[38].

While these observations clarify some points they also raise several new issues and questions. The most obvious one concerns nomenclature, where most researchers in the field, including the present authors, have improperly used the term subcutaneous to describe the adipocyte layer in rodent skin (for example [21],[26],[29]). Here we have shown that the skin adipose tissue originates entirely from within the dermis, and therefore suggest that it should, as some have already recognized [39],[1], be correctly called dermal adipose tissue. This terminology accurately reflects the anatomical origin of these cells as well as distinguishing them from other pre-established subcutaneous depots below the *panniculus carnosus*. We suggest that mouse dermal adipose tissue may indeed represent an adipose depot, distinct from subcutaneous white adipose tissue, and one whose molecular regulation is linked directly to skin and hair.

So when do the cells of the lower dermis destined to become adipocytes assume a different developmental fate from those of the upper dermis? FABP4 labelling in this study showed that the lower dermis was already different from the upper dermis at e16. However, the creation of a dense dermis occurs much earlier [40] and recent studies by the Atit group [41] link key cellular changes in the upper dermis around this period to Wnt signalling. Therefore, key differences between upper and lower dermis may be established at or around hair follicle initiation at e14.5 and it is possible that pre-adipocytes are present in the dermis at this time.

When discussing these issues it must be borne in mind that dermal cells from other body sites do not have the same embryonic origin. Ventral dermis originates from lateral plate mesoderm [42] while head dermis derives both from paraxial mesoderm and neural crest [43]. This latter point is interesting in the context of findings that a subset of facial adipocytes have neural crest origin [3].

There are very close links and reciprocal signalling between adipose tissue and the vascular system [44] that relate to the former’s role as an endocrine organ, the control of depot size, pathogenesis of adipose tissues, and even the onset of related diseases such as type 2 diabetes [45],[9]. During development, blood vessel formation is known to precede adipogenesis in some adipose depots [9] like in epididymal adipose tissue where there is evidence that angiogenesis can be mediated by macrophage activity [46]. Angiogenesis and adipogenesis have also been linked in subcutaneous adipose tissue [47]. Further work in our lab will aim to elucidate the relationship between adipocyte differentiation and capillary formation in skin dermis.

Of course not all other cells within late foetal and neonatal lower dermis cells are necessarily committed to adipogenesis and there may also be a hierarchy within the pre-adipogenic cells that includes a progenitor population. Related to this, within mature white adipose tissue (WAT) the adipose stroma has long been considered to be an important source of adipocyte stem cells [8]. A specific pre-adipocytic population was isolated from subcutaneous and parametrial white adipose tissue using flow cytometry and a panel of stem cell markers [48]. An equivalent population was more recently found to reside specifically within the mouse dermal adipose layer and display dynamic regenerative and signalling activity in concert with waves of hair follicle stem cell activity and cycling [27]. It would be interesting to investigate whether the precursors in mature skin have counterparts in the embryonic/foetal dermis?

The dynamic association between the thickness of the skin adipose layer and the hair cycle has long been recognized [22],[23],[24],[25]. In the current study we estimated the volume of lipid at the end of the first growth cycle (telogen) to be around a quarter of that at the middle of the growth phase (anagen). Previous reports have attributed fluctuations in the adipose layer to changes in size of the fat globules [25],[27] and not adipocyte cell numbers. However, a recent paper [27] discovered a burst of mitotic activity amongst progenitor cells in the adipose layer at a specific point in the follicle cycle. This implies that adipocyte cell numbers might increase over successive follicle growth cycles, or that if they remain constant, cell death is occurring. Germane to this, in our investigation we observed no significant apoptotic cell death in the adipose layer at any point in the first hair cycle.

### Conclusions and Further Questions

This study has demonstrated that the dermal adipose layer in the mouse develops from the lower dermis in a process that starts earlier in embryonic/foetal development than previously recognised and is intrinsic to the skin. In laboratory mice at least, the dermal adipose layer may therefore represent a separate depot from subcutaneous adipose tissue. The study also confirms a close spatio-temporal developmental link between hair follicles and adipocytes. These findings pose new questions, not least about when the upper and lower dermis become committed to different identities, what signalling is involved, and where it comes from? More work is also required to specifically unpick and track the lineage of dermal adipose tissue. Such investigations could also establish whether there are lineage relationships between dermal adipocytes and vascular tissues [5], and determine whether the blood supply acts as an intermediate in signalling between adipocytes and hair follicles? Genetically modified animals have helped researchers to implicate major signalling pathways in skin adipogenesis including Wnt [49] EGF [50], PDGF [51], [27] and BMP [52]. If dermal adipose tissue does indeed represent a different depot from subcutaneous white adipose tissue, further analysis should uncover specific molecular controls associated with its development and maintenance. Whether our findings have implications for adipose depots in the skin of other mammalian species remains to be seen? There are indications of species specificity in the molecular control of adipogenesis, for example in relation to the Wnt pathway [53]. However this study does impact on the mouse as a widely used experimental paradigm of skin and adipose development, homeostasis and disease.

## Supporting Information

Figure S1 **Images of Oil Red O stained skin with the selected areas artificially outlined and the contrast reduced in non-selected areas to highlight the distinction.** Three images from skin from six different individuals sacrificed at either 10 or 19 days.
(JPEG)

Figure S2 **A graph of the areas of Oil Red O staining of skin sections from six individuals sacrificed at 10 or 19 days.** Each set is the mean of three images from the same back skin sample. Error bars show the standard deviation. Ten day samples had 4.6 fold more lipid than 19 day ones, as determined by the area of Oil Red O staining - 319093 um^2^ (at 10 days) vs. 68531 um^2^ (at 19 days). The smallest observed ratio between any set was three fold greater at 10 days and the largest is eight fold.
(PNG)

Figure S3 **Fatty acid binding protein-4 (FABP4) expression is widespread within pre-adipocytes in the lower dermis from e16.** a) e15 (embryonic day 15), b) e16 (embryonic day 16), c) e18.5 (embryonic day e18.5). White dashed line delineates boundary between epidermis and upper dermis. Red dashed line delineates boundary between upper dermis and lower dermis. DNA was counterstained with 4′,6-diamidino-2-phenylindole (DAPI). Scale bar = 30 µm.
(PNG)

Table S1 **The average Oil Red O stained area of mouse back skin at 10 and 19 days.** Each set is a mean from three images from the same individual.
(PNG)
