# Correction: Development of the Mouse Dermal Adipose Layer Occurs Independently of Subcutaneous Adipose Tissue and Is Marked by Restricted Early Expression of FABP4

**DOI:** 10.1371/annotation/cf897d66-612e-42a7-a247-9979e4a61a8d

**Published:** 2013-10-09

**Authors:** Kamila Wojciechowicz, Karl Gledhill, Carrie A. Ambler, Craig B. Manning, Colin A. B. Jahoda

The second author was incorrectly indicated as the Corresponding Author for the article. In addition, the fifth author, Colin A.B. Jahoda, should be indicated as the Corresponding Author for the article. The fifth author's email address is: colin@jahoda@durham.ac.uk. 

