# Supplementary figures and images for: Development of the Mouse Dermal Adipose Layer Occurs Independently of Subcutaneous Adipose Tissue and Is Marked by Restricted Early Expression of FABP4
Source: PLoS One. 2013 Mar 26;8(3):e59811. doi: 10.1371/journal.pone.0059811 (PMC3608551; doi:10.1371/journal.pone.0059811)

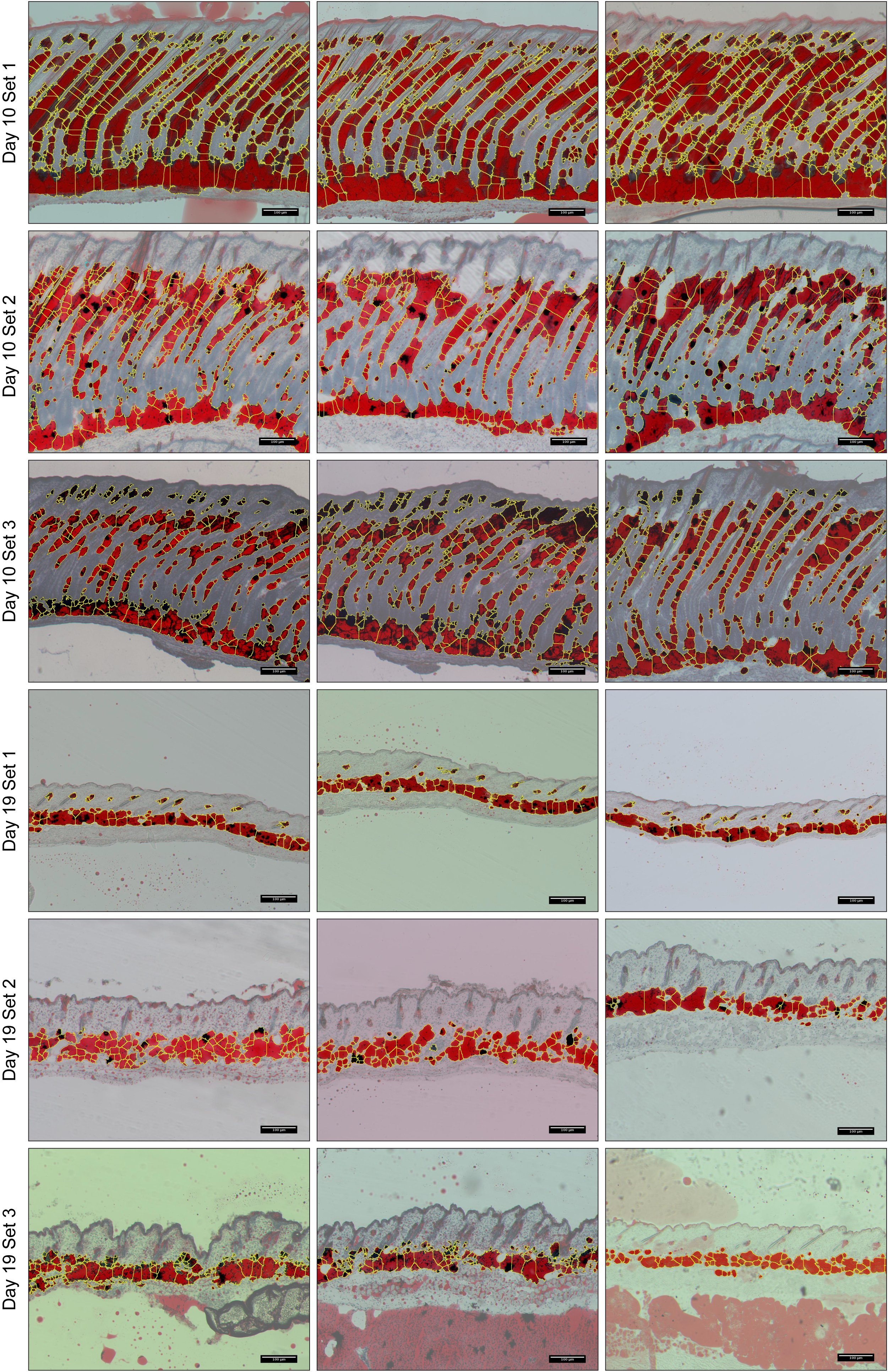

Supplement: Figure S1 — Images of Oil Red O stained skin with the selected areas artificially outlined and the contrast reduced in non-selected areas to highlight the distinction. Three images from skin from six different individuals sacrificed at either 10 or 19 days. (JPEG) [file pone.0059811.s001.jpeg]

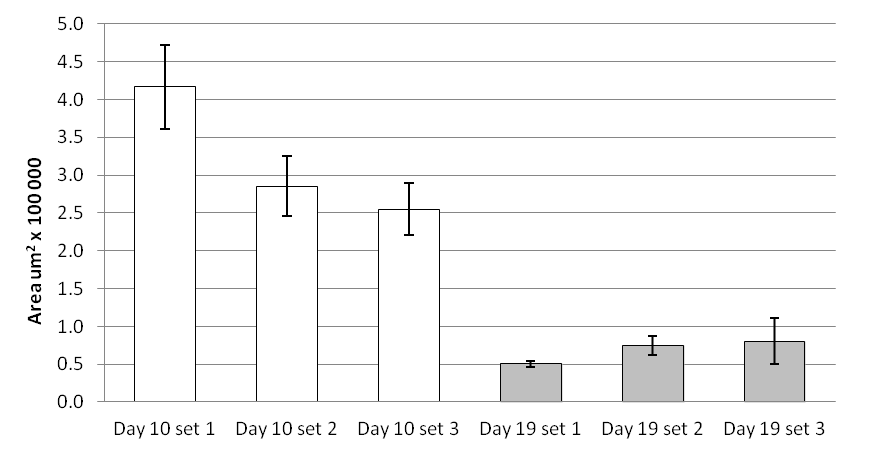

Supplement: Figure S2 — A graph of the areas of Oil Red O staining of skin sections from six individuals sacrificed at 10 or 19 days. Each set is the mean of three images from the same back skin sample. Error bars show the standard deviation. Ten day samples had 4.6 fold more lipid than 19 day ones, as determined by the area of Oil Red O staining - 319093 um2 (at 10 days) vs. 68531 um2 (at 19 days). The smallest observed ratio between any set was three fold greater at 10 days and the largest is eight fold. (PNG) [file pone.0059811.s002.png]

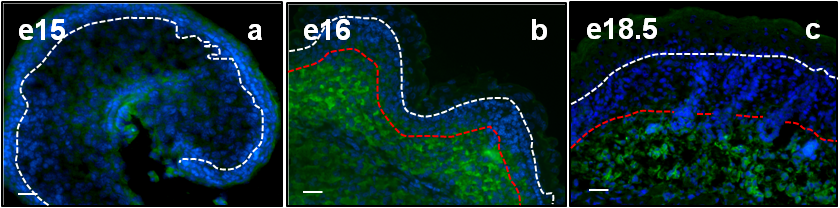

Supplement: Figure S3 — Fatty acid binding protein-4 (FABP4) expression is widespread within pre-adipocytes in the lower dermis from e16. a) e15 (embryonic day 15), b) e16 (embryonic day 16), c) e18.5 (embryonic day e18.5). White dashed line delineates boundary between epidermis and upper dermis. Red dashed line delineates boundary between upper dermis and lower dermis. DNA was counterstained with 4′,6-diamidino-2-phenylindole (DAPI). Scale bar = 30 µm. (PNG) [file pone.0059811.s003.png]

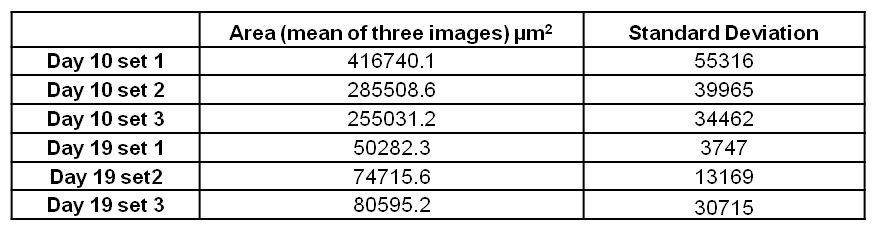

Supplement: Table S1 — The average Oil Red O stained area of mouse back skin at 10 and 19 days. Each set is a mean from three images from the same individual. (PNG) [file pone.0059811.s004.png]
